# Isolator-based point-of-care manufacturing: a practical solution for GMP-compliant cell and extracellular vesicles therapy production

**DOI:** 10.3389/fbioe.2025.1644318

**Published:** 2025-09-03

**Authors:** Yu-Sung Chiu, Kun-Liang Wang, Kun-Lieh Wu

**Affiliations:** ^1^ YJ. Biotechnology Co., Ltd., New Taipei City, Taiwan; ^2^ Department of Life Science, Fu-Jen Catholic University, New Taipei City, Taiwan; ^3^ Graduate Institute of Applied Science and Engineering, Fu-Jen Catholic University, New Taipei City, Taiwan; ^4^ Department of Electrical Engineering of I-Shou University, Kaohsiung, Taiwan

**Keywords:** cell therapy, extracellular vesicles (EVs), GMP-good manufacturing practice, isolator, point-of-care manufacturing

## Abstract

Cell and extracellular vesicle (EV)-based therapies represent a promising frontier in regenerative medicine and immunotherapy. However, their clinical translation is often constrained by the complexities of Good Manufacturing Practice (GMP)-compliant production, particularly under centralized manufacturing models. This Perspective discusses the emerging role of decentralized, point-of-care (POC) manufacturing in enabling timely, scalable, and patient-specific delivery of cell and EV therapeutics, with a focus on isolator-based systems as core manufacturing infrastructure. We discuss current advances in closed-system technologies, regulatory frameworks, and quality control (QC) strategies supporting GMP compliance in decentralized environments. Real-world applications and case studies illustrate feasibility and translational impact. Isolator-based platforms offer modular, sterile, automation-compatible environments that support both autologous and selected allogeneic product manufacturing at clinical sites. These systems reduce contamination risks, lower facility requirements, and enable integration with real-time QC testing. Despite these advantages, challenges remain, including regulatory ambiguity, workforce training limitations, and quality assurance gaps in decentralized settings. Emerging solutions include automated closed-system bioreactors, digitalized QC workflows, and harmonized operational standards to ensure product safety and consistency. Strategic coordination among regulators, hospitals, and developers will be essential to overcome operational and compliance hurdles. With appropriate infrastructure, skilled personnel, and standardized processes in place, isolator-based POC manufacturing holds the potential to transform how advanced therapies are produced and delivered—ultimately enhancing patient access to safe, effective, and personalized cell and EV-based treatments.

## Introduction

In recent years, the development of cell and gene therapies has progressed rapidly, offering new hope for treating a wide range of diseases with high unmet medical needs. Among these, non-genetically modified cell therapies and their derived extracellular vesicles (EVs) are particularly promising because of their lower regulatory complexity and potential for earlier clinical adoption (Wang et al., 2021; Bahmani and Ullah, 2022; Thom and Cronin, 2024).

To meet the growing demand, many contract development and manufacturing organizations (CDMOs) have expanded their capabilities to include the production of cellular and EV-based therapeutics. However, the successful clinical translation of these products critically depends on robust, scalable, and Good Manufacturing Practice (GMP)-compliant manufacturing systems (Patel et al., 2025). Key factors such as sterility assurance, process consistency, and batch reproducibility are essential to ensure product safety and efficacy (Stroncek et al., 2010).

Centralized manufacturing models, while well-established, present several logistical and technical limitations. Variables associated with transportation, including extended transit time, temperature fluctuations, and improper thawing procedures, can compromise the viability or biological activity of living cells and EV-based products (Lee and Chang, 2024; Gupta and Shaz, 2025). These challenges are particularly significant for Advanced Therapy Medicinal Products (ATMPs) (Pharmaceutical Inspection Co-operation Scheme (PIC/S), 2022), which are often administered shortly after culture or thawing and tend to be sensitive to cryopreservation (Meneghel et al., 2020; Joules et al., 2025).

Point-of-Care (POC) manufacturing has emerged as a compelling alternative to centralized production (Harrison et al., 2018; Lam et al., 2021). By enabling on-site manufacturing within or near the clinical setting, POC models minimize transportation risks, reduce time-to-treatment, and may allow for the administration of fresh, non-cryopreserved products. This approach may improve patient accessibility and streamline operational costs. Nonetheless, hospitals and clinics present inherent risks of contamination due to their high microbial burden and non-sterile environments. Since ATMPs cannot undergo terminal sterilization, rigorous aseptic processing and comprehensive quality control are essential (Harrison et al., 2017). Ensuring sterility remains one of the primary challenges in implementing POC manufacturing.

Isolator-based systems are widely employed in the pharmaceutical industry as sealed containment devices designed to physically separate operators from the manufacturing environment, thereby maintaining aseptic conditions (Levitan and Perry, 1968; Akers, 2015; Pharmaceutical Inspection Co-operation Scheme (PIC/S), 2007). These closed barrier systems can be classified into positive pressure isolators, typically used for handling sterile products to protect them from external contamination, and negative pressure isolators, used for handling hazardous substances to protect the operator. Within hospital-based POC manufacturing, positive pressure isolators are most commonly employed. These systems typically include integral glove ports, rapid transfer ports for material ingress and egress, integrated decontamination units (e.g., vaporized hydrogen peroxide (VHP) cycles, sporicidal decontamination), and independent heating, ventilation, and air conditioning (HVAC) filtration modules, enabling ISO Class 5 environments within non-classified hospital rooms (Krishna et al., 2000; International Organization for Standardization (ISO), 2004).

The choice of isolator configuration depends on process requirements, product risk profiles, and institutional infrastructure. Their modularity and relative ease of installation compared to traditional cleanrooms make them particularly attractive for decentralized ATMP manufacturing in clinical settings (Kokubo et al., 2016). Unlike biological safety cabinets (BSCs), which provide open-front airflow protection within classified cleanrooms, isolators are completely closed systems that maintain asepsis independent of the surrounding environment. Compared to restricted access barrier systems (RABS), which offer partial separation and rely on the background cleanroom for contamination control, isolators provide a fully sealed workspace with integrated decontamination and do not require installation within high-grade cleanroom environments. This fundamental difference makes isolators particularly advantageous for POC manufacturing in hospitals and clinics where full GMP cleanroom infrastructures are often unavailable.

By enabling the entire workflow—from tissue acquisition and cell processing to final formulation and reinfusion—to be carried out within a controlled, closed environment at or near the patient’s bedside, isolator-based platforms reduce supply chain complexity, enhance sterility assurance, and preserve the biological integrity of sensitive ATMPs (Savry et al., 2014).

In this Perspective, we explore the integration of GMP-compliant manufacturing strategies for cell and EV-based therapies, with particular emphasis on POC production models and the role of isolator-based systems as core enabling technologies.

## Therapeutic applications of cell and EV therapies

Cell and EV therapies can be broadly categorized into two classes: immune effector cell-based and regenerative/stromal cell-based. Each category presents unique therapeutic applications and inherent challenges and is being explored across a wide range of clinical indications.

### Immune cell-based therapies

Immune cell-based therapies, including cytokine induced killer (CIK) cells (Schmeel et al., 2015), natural killer (NK) cells (Wu et al., 2020), dendritic cells (DCs) (Li et al., 2009), and gamma-delta (γδ) T cells (Arias-Badia et al., 2024), have demonstrated promising antitumor activity in cancer immunotherapy (Takimoto et al., 2023; Toner et al., 2024). These cells are often used to directly eliminate malignant targets or to boost the host’s immune response.

A major advantage of immune effector cells such as CIK cells, NK cells and γδ T cells lies in their non-major histocompatibility complex (MHC)-restricted cytotoxicity. Their antitumor activities do not rely on classical antigen presentation pathways (Rees, 1990; Maniar et al., 2010), facilitating the development of off-the-shelf, allogeneic immunotherapies that can be administered to multiple patients without the need for human leukocyte antigen (HLA) matching or patient-specific sourcing. Moreover, their efficacy can be augmented through combination with antibody-based therapeutics. NK cells and γδ T cells mediate antibody-dependent cellular cytotoxicity (ADCC) through their surface CD16 receptors when engaged by monoclonal antibodies targeting tumor-associated antigens (Wang et al., 2008; Junttila et al., 2010). Vγ9Vδ2 γδ T cells exhibit synergistic cytotoxicity when combined with bispecific T cell engagers (BiTEs) such as blinatumomab, which redirects T cell activity toward malignant B cells (Chen et al., 2021). These combinatorial approaches broaden the clinical applicability of non-MHC-restricted effector cells and reinforce their potential as scalable, allogeneic immunotherapies in oncology.

### Regenerative and stromal cell-based therapies

In contrast, mesenchymal stromal cells (MSCs),are primarily being investigated for the treatment of chronic and degenerative diseases such as osteoarthritis (Copp et al., 2023; Cao et al., 2025), Parkinson’s disease (Heris et al., 2022), and chronic kidney disease (CKD) (Zheng et al., 2022), as well as for immunomodulation in systemic lupus erythematosus (SLE) (Li et al., 2021; Farge et al., 2025) and graft-versus-host disease (GvHD) (Che et al., 2020; Kadri et al., 2023; Blanc et al., 2025). MSCs can be sourced from various tissues, including bone marrow (BMSCs), adipose tissue (ADSCs), and perinatal tissues such as Wharton’s jelly (WJ-MSCs), each offering distinct advantages in terms of accessibility, immunomodulatory potential, and scalability (Hass et al., 2011).

MSCs are often considered immune-privileged due to their low expression of MHC class I and II molecules, which reduces immune recognition and rejection. This property, combined with their inherent immunosuppressive effects, underlies their therapeutic utility in treating inflammatory and autoimmune conditions (Petrus-Reurer et al., 2021; Oh et al., 2022).

### Extracellular vesicle therapies

A notable advancement is the emergence of EVs, particularly exosomes (Exo) derived from stem cells, as a novel class of cell-free therapeutics. Regarded by some as the foundation of “Cell Therapy 2.0,” Exo can replicate many biological effects of their parent cells while avoiding risks such as immunogenicity, tumorigenicity, and engraftment failure (Rohde et al., 2019; Lai et al., 2022; Williams et al., 2022). Their nanoscale size and ability to carry bioactive molecules including proteins, lipids, and nucleic acids make them attractive candidates for regenerative medicine, immunomodulation, and drug delivery applications.

### Autologous vs. allogeneic therapies

Cell therapies typically involve the administration of either autologous (patient-derived) or allogeneic (donor-derived) cells, expanded or manipulated *ex vivo* before reinfusion to exert therapeutic effects (Chen et al., 2022). Allogeneic therapies require stringent immunological and functional criteria to minimize graft-versus-host or host-versus-graft responses (Deuse and Schrepfer, 2025). NK cells, γδ T cells, and MSCs are particularly attractive for allogeneic use due to their immunological properties and low risk of eliciting strong host immune responses (Petrus-Reurer et al., 2021; Oh et al., 2022).

### Genetically engineered cell therapies

Genetically engineered therapies, including chimeric antigen receptor T (CAR-T) cells and induced pluripotent stem cell (iPSC)-derived products, represent a major direction for future therapeutic development (Xue et al., 2023).

Among immune cell therapies, αβ T cells, particularly CAR-T cells, represent the most clinically advanced and widely approved approach. CAR-T cell therapies have demonstrated remarkable efficacy in hematologic malignancies, with approved products targeting relapsed or refractory B-cell acute lymphoblastic leukemia, diffuse large B-cell lymphoma, mantle cell lymphoma, and, more recently, B-cell maturation antigen (BCMA)-positive multiple myeloma (Ahmad et al., 2020; Perales et al., 2022; Wu and Dhakal, 2023). The manufacturing process involves autologous leukapheresis, genetic modification (typically via viral transduction) to express tumor-specific CAR constructs, *ex vivo* expansion, and rigorous quality control prior to reinfusion. Despite their clinical success, CAR-T therapies remain limited by complex, time-consuming, and high-cost manufacturing, as well as potential adverse effects such as cytokine release syndrome and neurotoxicity (Balagopal et al., 2022; Gatto et al., 2023).

In comparison, NK cells and γδ T cells offer simpler manufacturing without genetic modification, non-MHC-restricted cytotoxicity enabling allogeneic off-the-shelf use, and lower safety concerns, and, when combined with therapeutic antibodies, more precise targeting without genetic engineering. Nonetheless, the prominence of CAR-T cells underscores the transformative impact of T cell engineering in immuno-oncology and highlights the potential synergy between genetic modification and innate-like immune cell platforms. Notably, the development of CAR-engineered NK cells (CAR-NK) and CAR-engineered γδ T cells (CAR-γδ) is an emerging direction, combining the advantages of innate immune effector functions with targeted recognition, thereby expanding the future therapeutic landscape of adoptive cell therapies (Xian and Wen, 2025; Marofi et al., 2021).

Continued progress in gene editing, automated manufacturing, and safety engineering is progressively overcoming technical and regulatory barriers, expanding the clinical potential of these advanced modalities.

## Advantages of decentralized and POC manufacturing

To ensure batch-to-batch consistency in cell and EV-based therapies, each newly produced batch is typically evaluated against prior preparations using biochemical, biophysical, and functional assays (Rohde et al., 2019). The integrity and reproducibility of these comparisons are especially critical when transitioning from research-grade to clinical-grade manufacturing, where regulatory standards demand rigorous validation.

Autologous cell therapies, including autologous chondrocytes, BMSCs, ADSCs, and immune cell products such as CIK cells and DCs, are typically non-genetically modified, patient-specific, and manufactured in small batches (Marlovits et al., 2006; Wermke et al., 2025). These therapies often possess short therapeutic windows and limited cryostability, making fresh, on-demand production particularly advantageous. Consequently, POC manufacturing is especially suited to these modalities by enabling onsite production within or near the clinical setting. This arrangement significantly reduces transportation time, simplifies logistics, and shortens the overall vein-to-vein interval (Harrison et al., 2017), which is critical for preserving cell viability and therapeutic potency.

In contrast, cell and EV therapies intended for allogeneic use—such as NK cells, γδ T cells, MSCs, and stem cell-derived EVs—are designed for administration to multiple patients. These products can be manufactured at scale, cryopreserved, and distributed as off-the-shelf therapeutics. Centralized manufacturing remains the dominant approach for such therapies due to its economies of scale and well-established GMP infrastructure (ten Ham et al., 2020). However, in hospitals with high patient throughput, establishing in-house production facilities may offer both clinical and operational advantages by ensuring consistent supply, reducing reliance on external CDMOs, and enabling rapid adaptation to local clinical needs. In such contexts, decentralized manufacturing models can be adapted to support routine and localized delivery of allogeneic therapies (Harrison et al., 2018; Lam et al., 2021).

Gene-modified therapies, including iPSC-derived products and CAR-T cells, present distinct biosafety and technical challenges for POC implementation (Nakagawa et al., 2014; Moradi et al., 2023). The manufacturing process for these therapies involves genetic manipulation steps, such as viral transduction or non-viral gene editing, which require stringent containment to prevent vector leakage and ensure operator safety (Marofi et al., 2021; Abou-El-Enein et al., 2021; Rodrigues et al., 2021). Nevertheless, isolator-based manufacturing platforms can support the safe handling of both viral and non-viral gene transfer technologies. These closed systems allow critical steps, gene transduction, cell expansion, and final formulation, to be performed within a fully contained, GMP-compliant environment, thereby minimizing environmental contamination risks and making onsite production of certain gene-modified therapies technically viable.

**FIGURE 1:**
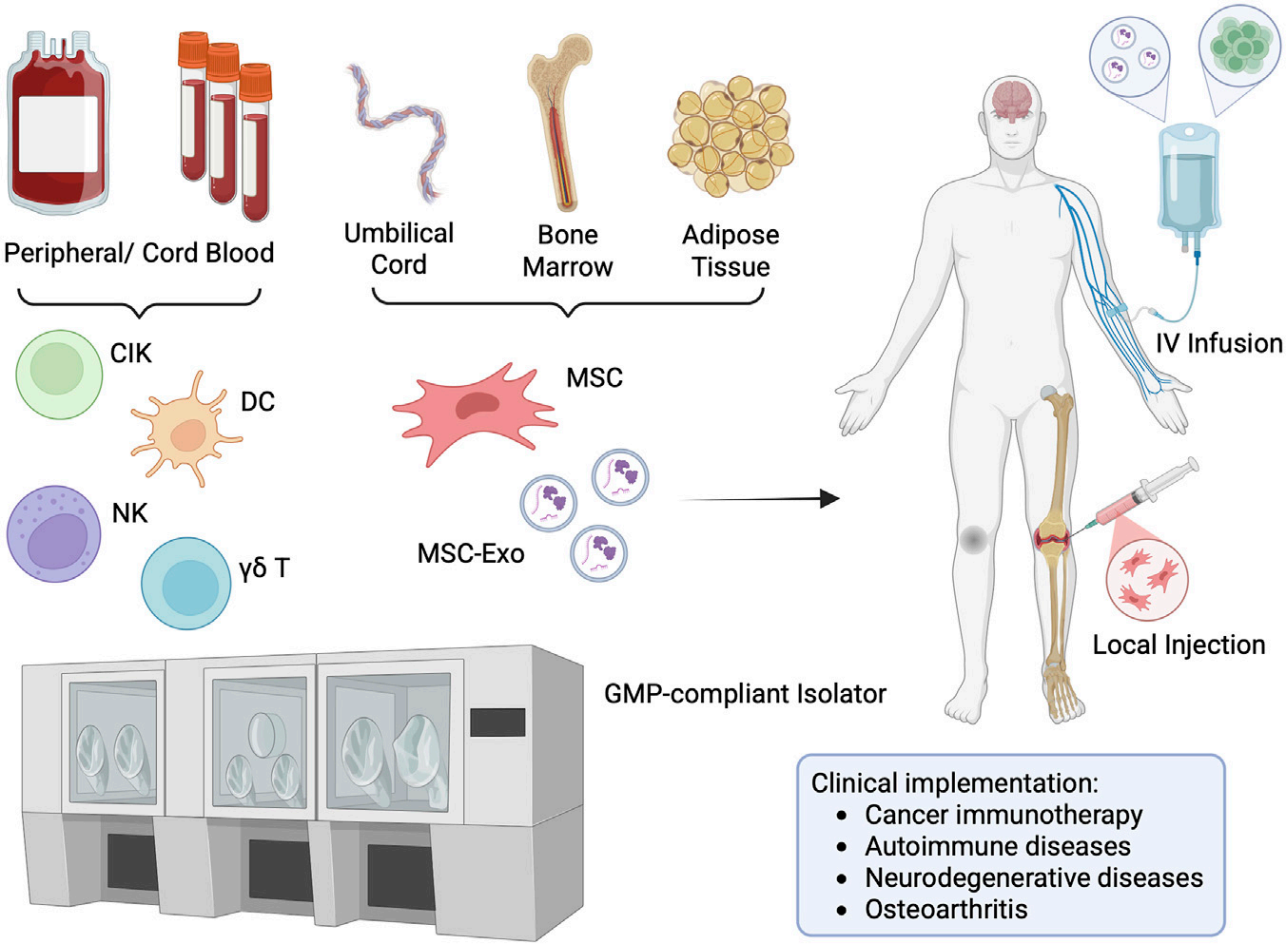
Isolator-Based POC Manufacturing for Cell/EV Therapies. Legend: Schematic representation of point-of-care manufacturing using an isolator system. Cells are first isolated from donor or patient tissue, then expanded and processed within a closed, GMP-compliant isolator in the hospital. This enables on-site production of immune or stem cell-based therapies and immediate administration back to patients. The entire workflow—from tissue extraction, cell isolation, expansion, formulation, quality control, to final infusion—can be seamlessly integrated and completed within the hospital-based isolator system. This end-to-end, closed-system approach minimizes contamination risk, ensures regulatory compliance, and significantly reduces time between manufacturing and patient treatment. Figure was created in BioRender. Chiu, YS (2025) https://BioRender.com/ph65tdh.

Overall, isolator-based POC platforms offer modularity, scalability, and a high degree of contamination control, making them enabling technologies for distributed manufacturing of both autologous and selected allogeneic therapies (Figure 1). These systems not only reduce reliance on centralized facilities but also broaden access to advanced, personalized, and gene-modified therapies in hospital or regional care settings.

## Isolator-based systems as core manufacturing infrastructure

Recent technological advances have led to the development of modular isolator-based platforms tailored for decentralized, hospital-based use. These closed systems integrate cell expansion, harvesting, and final formulation within a single sterile unit, offering a streamlined approach to manufacturing ATMPs near the point of care (Harrison et al., 2017; Shah et al., 2023).

In the context of GMP-compliant cell and EV production, contamination control is paramount. Traditional aseptic technologies, such as BSCs and RABS, offer only partial protection and typically require installation within Class B cleanrooms featuring validated airflow patterns and stringent environmental controls (Pharmaceutical Inspection Co-operation Scheme (PIC/S), 2023). In contrast, isolator systems are engineered as fully enclosed, sealed environments that can operate in less stringent background conditions, such as Grade C or D clean areas, without compromising sterility (Table 1) (Pharmaceutical Inspection Co-operation Scheme (PIC/S), 2023; Pharmaceutical Inspection Co-operation Scheme (PIC/S), 2022). This capability results from their compliance with international containment and leak-tightness standards, including ISO 14644-7 (International Organization for Standardization (ISO), 2004) and ISO 10648-2 (International Organization for Standardization (ISO), 1994).

**TABLE 1:** Comparison of BSC, RABS and Isolator Systems for Containment and Decontamination. While BSCs and RABS rely on manual disinfection and operate within a Grade B cleanroom (ISO 5 at rest, ISO 7 in operation) with high air change rates, isolators offer a fully enclosed environment with automated decontamination, validated leak control, and can operate in a lower-grade background (Grade D, ISO 8) due to their inherent containment efficiency. These characteristics make isolators particularly suitable for high-containment applications and standardized GMP-compliant manufacturing. Air Change Rate (ACH) stands for Air Changes per Hour. It is a measure of how many times the entire volume of air in a defined space is replaced with fresh or filtered air within 1 hour.

Criteria	BSC	RABS	Isolator
Decontamination	Manually disinfected	Manually disinfected	Quantifiable and highly reproducible method via automated bio-decontamination
Assurance of Separation	No physical separation from surrounding environment	Partial physical separation; integrity depends on operator compliance	Fully closed system with validated leak rate and continuously monitored differential pressure
Surrounding Environment	Grade B In Rest: ISO 5 In operation: ISO 7	Grade B In Rest: ISO 5 In operation: ISO 7	Grade D ISO8 (both at rest and in operation)
Air Change Rate (ACH)	High (ISO 5: ≥240–300 ACH)	High (ISO 5: ≥240–300 ACH)	Lower (ISO 8: ∼20 ACH), as the isolator provides primary containment
Toxic Containment	Low Capability	Moderate Capability	High reliability and safety for toxic or hazardous materials

By maintaining a closed and controlled environment, isolator-based systems significantly reduce reliance on the complex HVAC infrastructure necessary for conventional cleanroom manufacturing. This design ensures robust sterility assurance and process containment, which are critical for the safe production of ATMPs that cannot undergo terminal sterilization.

Isolators provide several critical advantages for POC applications. First, they minimize human intervention and environmental exposure, significantly reducing the risk of microbial contamination. Second, they serve as a cost-effective infrastructure option by lowering cleanroom classification requirements, thereby reducing facility construction and operational costs. Third, isolator systems can be easily integrated with automated culture, monitoring, and filling technologies, enhancing both process standardization and scalability.

For small and medium-sized biotech companies or academic institutions aiming to meet GMP standards, isolator-based systems present a practical solution. However, the full benefit is realized only when the entire manufacturing workflow is maintained within a closed and validated process chain. This includes steps such as cell cultivation, EV enrichment or purification, and final fill-finish. Under these configurations, isolators not only ensure sterility assurance but also provide a flexible and modular platform capable of adapting to the evolving needs of clinical manufacturing.

Indeed, isolator-based technologies have revolutionized aseptic processing over the past two decades by effectively separating human operators from the production environment, thereby dramatically reducing contamination risks and enabling the safe manufacture of heat-labile or sensitive biological products (Kokubo et al., 2016).

For example, in reproductive medicine, the implementation of fully enclosed isolator systems for *in vitro* fertilization (IVF) has significantly improved embryo development to the blastocyst stage, yielding higher cell numbers and accelerated growth compared to conventional open-fronted laminar flow hoods, ultimately resulting in increased pregnancy and implantation rates (Hyslop et al., 2012).

Our previously published study demonstrated the therapeutic use of EVs derived from human WJ-MSCs manufactured under GMP conditions within an isolator-based system for treatment in a rodent model of ischemic stroke. The resulting exosomes exhibited significant neuroprotective effects *in vitro* and *in vivo*, underscoring how isolator-based systems can facilitate the production of clinically applicable EVs with consistent quality and safety (Chiu et al., 2024).

Beyond these examples, isolator-based closed systems are increasingly applied in cell therapy manufacturing to address sterility and cost challenges inherent to conventional cleanroom-dependent production. For instance, the Tissue Factory, developed as the first practical implementation of the flexible modular platform, integrates multiple automated modules within biologically sealed isolator chambers decontaminated by VHP. This system successfully manufactured multi-layered skeletal myoblast sheets, expanded human articular chondrocytes, and cultured human iPSCs across multiple passages without microbial contamination, demonstrating robust performance comparable to manual operations while minimizing operator intervention and contamination risks. Such automated isolator-based platforms represent a paradigm shift towards scalable, cost-effective, and standardized production of cell-based healthcare products (Kikuchi et al., 2018).

Moreover, as highlighted by recent manufacturing facility design analyses, traditional open system production in Grade B cleanrooms is being reconsidered in favor of isolator-based closed systems. Isolators, operating as Grade A environments within at least Grade D cleanroom backgrounds, provide superior sterility assurance and operator safety. They also reduce facility operational complexity and cost, enabling reproducible GMP-compliant manufacturing of ATMPs at larger scales. Consequently, isolator workstations are now considered viable and attractive alternatives to classical cleanrooms, offering flexible, compact, and cost-efficient solutions to meet stringent regulatory requirements while maintaining high product quality and safety standards (Zanini et al., 2020).

In summary, isolator-based systems represent a cornerstone technology for the implementation of decentralized, GMP-compliant biomanufacturing. Their integration into POC models has the potential to democratize access to cell and EV therapies by lowering barriers to clinical-grade production and enabling safer, more efficient delivery at the patient’s bedside.

## Quality control and regulatory considerations for GMP-compliant POC biomanufacturing

In cell- and EV-based therapies, where the “process is the product,” maintaining strict quality control (QC) is critical to ensuring safety, consistency, and therapeutic efficacy (Rohde et al., 2019). This is especially true for decentralized POC manufacturing models, where reduced shelf-life and limited batch sizes demand real-time release strategies and in-process validation. GMP compliance requires implementation of pharmacopeia-aligned QC strategies, including sterility, mycoplasma, and endotoxin testing, alongside rigorous product characterization (Council of Europe, 2023; United States Pharmacopeial Convention (USP), 2023).

### Sterility testing

Sterility is a mandatory release criterion for all injectable ATMPs. Traditionally, compendial sterility testing requires a 14-day incubation period, delaying product release and posing logistical challenges for fresh, non-cryopreserved therapies. Recent advancements have enabled rapid sterility testing methods to accelerate release timelines.

For example, automated fluorescence-based microbial detection systems, such as BacT/Alert, detect metabolic activity or CO_2_ production of microorganisms in culture bottles, providing results within 3–5 days rather than 14 days (Lim et al., 2014; Bugno et al., 2015). Alternatively, direct fluorescent staining and membrane filtration approaches, as employed in systems like ScanRDI, enable near real-time detection by capturing microorganisms on a membrane, staining with nucleic acid-binding fluorophores, and enumerating them via laser scanning cytometry, reducing detection time to less than 3 h depending on bioburden levels (Smith et al., 2010; Mohammadi et al., 2023).

Such automated microbial detection systems are already widely used in hospitals, which can facilitate the adoption of POC manufacturing at clinical sites. Combining these rapid sterility tests with isolator-based closed manufacturing environments can minimize patient and manufacturing risk by enabling timely product release while maintaining stringent sterility assurance.

### Mycoplasma testing

Mycoplasma are among the smallest prokaryotic organisms and pose a persistent threat to cell culture systems due to their intracellular parasitic nature, lack of a cell wall, and resistance to standard antibiotics (Totten et al., 2023). Their small size and absence of visible effects make them undetectable through routine microscopic inspection. Although EV-based therapies are acellular, the upstream use of primary cells and animal-origin reagents during culture introduces a significant risk of contamination. Therefore, *mycoplasma* testing should be conducted after cell expansion and before EV isolation to ensure downstream product safety. qPCR-based *mycoplasma* detection assays have largely replaced traditional culture methods due to their ability to deliver validated results within 1–4 h, supporting rapid batch release in POC settings (Becherucci et al., 2021).

### Endotoxin testing

Endotoxins are lipopolysaccharide (LPS) components of Gram-negative bacteria that can trigger severe immune responses if administered intravenously. Regulatory agencies impose strict endotoxin limits for both systemic and localized administrations. The limulus amebocyte lysate (LAL) assay remains the gold standard for endotoxin detection and must be validated for use with each product type and formulation (Gorman, 2020). Rapid turnaround is particularly critical in POC settings to enable timely release. The use of pyrogen-free materials and closed-system fluidics within isolators further reduces endotoxin contamination risks.

### Cell characterization

Cell identity, purity, and potency must be assessed prior to release. For immune cell populations, broad lineage distribution is initially evaluated using CD45 for pan-leukocyte gating, CD3 for total T cells, CD4 and CD8 for T cell subsets, CD19 for B cells, CD16 and CD56 for NK cells, and CD14 for monocytes, providing a general immunophenotypic overview before detailed subset analysis (Apoil et al., 2017).

For CIK cells, characterization typically includes immunophenotyping for CD3^+^ T cell lineage, CD56^+^ NK-like subsets, and the CD3^+^CD56^+^ NKT cell population, which mediates major cytotoxicity (Schmidt-Wolf et al., 1993; Linn et al., 2009; Liu et al., 2017). DCs are identified by high expression of CD11c and HLA-DR, with co-stimulatory molecules such as CD80 and CD86 used to confirm identity and maturation status (Szabolcs et al., 1996; Liu et al., 2009; Collin and Bigley, 2018). NK cell products are defined by a minimum phenotypic standard of CD56^+^CD3^−^ to confirm NK lineage, with CD45 positivity confirming leukocyte identity. CD16 expression is variably documented to distinguish functional subsets (Koehl et al., 2016; Wagner et al., 2019; Jahan et al., 2024). γδ T cells are identified by T cell receptor (TCR) γδ expression, distinguishing them from conventional αβ T cells. They typically co-express CD3 as part of the TCR complex, while CD45RA and CD27 are used for further subset characterization into naïve, central memory, effector memory, and terminally differentiated phenotypes (Yazdanifar et al., 2020; Chen et al., 2021; Barros-Martins et al., 2022; Sanz et al., 2023).

Potency assays for these immune effector cell types primarily evaluate cytotoxic activity against tumor or pathogen-infected cell lines using either traditional radioactive chromium-51 release assays (Elsner and Dressel, 2020), non-radioactive flow cytometry-based killing assays (Fischer and Mackensen, 2003), or luminescence-based assays that measure target cell viability through ATP quantification or other metabolic readouts (Contag and Bachmann, 2002; Karimi et al., 2014).

For MSCs, the International Society for Cell and Gene Therapy (ISCT) outlines three defining criteria: (1) plastic adherence under standard culture conditions, (2) expression of surface markers CD105, CD73, and CD90, with negative expression of hematopoietic markers (e.g., CD45, CD34, CD14/CD11b, CD79α/CD19, HLA-DR), and (3) trilineage differentiation potential into osteoblasts, adipocytes, and chondrocytes (Dominici et al., 2006).

These identity and potency assessments serve as critical release criteria to ensure that each cell product meets defined quality and functional specifications prior to clinical administration.

### EV characterization

EVs are characterized according to the MISEV2018 guidelines published by the International Society for Extracellular Vesicles (ISEV) (Théry et al., 2018). Given that EV preparations often contain a heterogeneous mix—including exosomes, microvesicles, and apoptotic bodies—proper classification and batch-to-batch consistency are essential. Key parameters include: (1) Size and morphology (typically 30–150 nm) via nanoparticle tracking analysis (NTA) or transmission electron microscopy (TEM). (2) Marker profiling, including tetraspanins (CD63, CD81, CD9) and exclusion of non-EV proteins (e.g., GM130, Calnexin) (3) Cargo analysis, such as protein and RNA content via Western blotting, ELISA, or sequencing. Although “exosomes” are often used as a general term, their strict definition implies endosomal origin. Without specific biogenetic validation, the term “small EVs” is preferred (Welsh et al., 2024).

By combining isolator-based closed-system manufacturing with in-hospital QC testing, POC models can ensure timely release of safe, GMP-compliant cell and EV products. This approach minimizes contamination risks, supports real-time decision-making, and reduces the logistical burdens of centralized manufacturing. As decentralized production gains traction, robust QC strategies will remain central to ensuring consistent quality and clinical efficacy of these next-generation therapies.

## Discussion

Despite its transformative potential, POC manufacturing of cell- and EV-based therapies faces significant regulatory, operational, and technical hurdles. These challenges must be addressed to enable the safe, scalable, and sustainable implementation of POC manufacturing within clinical settings.

Cell-based therapies often straddle the boundary between pharmaceutical products and medical procedures, a classification with substantial regulatory implications. If considered a medical procedure, the therapy may fall under medical practice regulations with limited oversight (Blanc et al., 2025). Conversely, classification as a medicinal product—particularly as an ATMP—triggers compliance with full pharmaceutical inspection convention and co-operation scheme (PIC/S) GMP standards, sterility assurance requirements (Pharmaceutical Inspection Co-operation Scheme (PIC/S), 2023), and formal product release testing (Pharmaceutical Inspection Co-operation Scheme (PIC/S), 2022; Ilic et al., 2012a; Ilic et al., 2012b). This ambiguity poses a fundamental question: Can hospital-based sites legally manufacture and administer such therapies, or must this remain within the domain of licensed pharmaceutical manufacturers?

In some jurisdictions, the “hospital exemption” pathway allows for non-commercial use of ATMPs within the same institution under defined conditions, facilitating certain POC manufacturing models (Sánchez-Guijo et al., 2023). In parallel, expanded access programs (EAPs) provide a regulatory mechanism for compassionate use of investigational therapies in patients lacking alternative treatment options (CellTrials, 2024). While HE typically focuses on treatments prepared and used within the same hospital, EAPs can enable broader access to investigational products across institutions prior to formal market approval (Klopfenstein et al., 2015; Jarow et al., 2017). Isolator-based POC manufacturing directly supports both HE and EAP frameworks by enabling decentralized, small-scale production of ATMPs or EV therapies under GMP-compliant conditions within hospital settings. In HE pathways, this approach facilitates in-hospital preparation and immediate clinical use without centralized manufacturing. In EAP contexts, where rapid provision of investigational therapies is critical for patients without alternatives, isolator-based POC enables on-demand preparation of personalized products while ensuring regulatory-required quality standards, thus bridging operational feasibility and compassionate use regulatory frameworks. However, regulatory alignment across countries remains inconsistent, complicating international deployment and harmonized practice standards.

Beyond regulatory ambiguity, limitations in workforce availability and technical expertise remain major barriers to POC manufacturing implementation. Effective operation requires multidisciplinary teams proficient in aseptic processing, clinical-grade reagent handling, and QC testing. Operators must be trained to perform or supervise critical processes such as sterility assurance, mycoplasma testing, endotoxin screening, and advanced analytics for cell and EV characterization. However, the availability of such skilled personnel is often limited, particularly in smaller clinical centers, and maintaining operator proficiency within GMP frameworks introduces significant training and documentation burdens.

Strategies to overcome these challenges include not only centralized training programs, competency certification schemes, and vendor-supported technical training modules to ensure consistent operational quality across sites, but also implementation of centralized cloud-based manufacturing execution systems (MES) and electronic batch record (eBR) platforms. These digital systems enable real-time data collection, monitoring, and cross-site analysis, ensuring consistent process parameters, environmental monitoring, and product release criteria across decentralized sites. Additionally, centralized quality assurance (QA) oversight structures can review and harmonize batch release decisions, while standardized digital standard operating procedures (SOPs) further enforce procedural uniformity. Integration of isolator operations within these electronic systems, compliant with FDA 21 CFR Part 11 requirements for electronic records and signatures, ensures that all processing and environmental monitoring data are securely captured, traceable, and fully auditable (Food and Drug, 1997). Together, these approaches directly address the challenges of standardization and centralized data monitoring, facilitating robust quality assurance across diverse clinical manufacturing settings.

Infrastructure and automation remain additional constraints on scalability. While isolator-based systems reduce reliance on traditional cleanrooms, their setup and operation still require stringent environmental controls, validated decontamination procedures, and ongoing maintenance. Furthermore, the availability of isolator systems within clinical centers is often limited by high capital costs, spatial requirements, and maintenance demands, particularly outside large academic hospitals. Emerging automated, closed-system platforms—such as robotic cell culture systems and automated filling units—offer promising alternatives by minimizing human handling, reducing contamination risks, and increasing batch-to-batch consistency (Gupta and Shaz, 2025; Palani et al., 2023; Trainor et al., 2023). Integrating robotic arms within isolator systems represents a promising hybrid approach, enabling high-throughput, GMP-compliant cell manufacturing in compact, modular setups (Melocchi et al., 2024; Ochs et al., 2022).

However, these technologies remain costly and are not yet widely adopted in clinical practice. Initiating implementation at major academic hospitals, where technical expertise, infrastructure, and regulatory support are more accessible, represents a viable starting point. Broader adoption could be facilitated through standardized protocols, prequalified GMP-grade materials, and modular infrastructure designs tailored for hospital pharmacies. To overcome financial barriers, cost-sharing models such as interdepartmental equipment utilization, regional manufacturing hubs, public–private partnerships, or vendor-supported leasing and pay-per-use models may be necessary, especially within publicly funded healthcare systems.

QA and regulatory compliance in decentralized settings pose further challenges. Centralized manufacturing facilities typically maintain dedicated QA departments and well-established SOPs, whereas decentralized POC sites may lack extensive experience implementing full GMP-compliant QA systems, increasing the risk of inconsistent product quality or regulatory non-compliance (Shah et al., 2023). Establishing harmonized SOPs across sites, adopting digital batch records with centralized data monitoring, and enabling remote QA oversight, such as cloud-based audits or centralized review of production and release data, could mitigate these risks while ensuring consistent quality standards across multiple clinical settings (Harrison et al., 2018; Harrison et al., 2017).

While automation and isolator-based manufacturing technologies have begun to lower some barriers, widespread adoption of POC manufacturing for cell and EV therapies will require harmonized regulations, targeted workforce development, standardized operational protocols, and continued innovation in GMP-compatible bioprocessing systems. Collaborative efforts among regulators, hospitals, academia, and industry stakeholders will be essential to translate this promising manufacturing model into routine clinical practice, ultimately broadening patient access to safe, effective, and personalized advanced therapies.

## Conclusion

The field of cell and EV therapies is advancing rapidly, driven by increasing clinical demand, emerging biotechnologies, and growing recognition of their therapeutic potential across a broad spectrum of diseases. Traditional manufacturing models are shifting away from centralized, large-scale facilities toward more flexible and decentralized POC strategies.

Innovations such as isolator-based systems, closed-system automation, and artificial intelligence-assisted quality control are redefining how GMP-compliant production can be achieved within hospital or near-patient settings. Concurrently, the development of allogeneic therapies and off-the-shelf biologics introduces new opportunities for scalability, logistical efficiency, and regulatory standardization.

To ensure the safe, effective, and equitable implementation of these therapies, close collaboration among regulators, manufacturers, clinicians, and academic researchers is essential. Harmonizing international guidelines, expanding workforce training programs, and investing in isolator-based, automated platforms will be critical to realizing the full potential of decentralized, GMP-compliant production of next-generation cell and EV therapies.

Ultimately, the vision is to establish robust and compliant infrastructures that support timely, patient-specific treatments without compromising safety or quality. POC manufacturing, supported by isolator technology and automation, offers a promising pathway to making advanced therapies more accessible while maintaining the stringent standards necessary for clinical success.

## Glossary

ACH: Air Change Rate
ADCC: Antibody-Dependent Cellular Cytotoxicity
ADSC: Adipose-Derived Stromal Cell
ATMPs: Advanced Therapy Medicinal Products
ATP: Adenosine Triphosphate
BiTE: Bispecific T cell Engagers
BMSC: Bone Marrow-derived Stromal Cell
BSC: Biological Safety Cabinet
CAR-T: Chimeric Antigen Receptor T
CDMOs: Contract Development and Manufacturing Organizations
CIK: Cytokine-Induced Killer
CKD: Chronic Kidney Disease
EAPs: Expanded Access Programs
eBR: Electronic Batch Record
EV: Extracellular Vesicle
GMP: Good Manufacturing Practice
GvHD: Graft-versus-Host Disease
HLA: Human Leukocyte Antigen
HVAC: Heating, Ventilation, Air-conditioning and Cooling
iPSC: Induced Pluripotent Stem Cell
ISCT: International Society for Cell and Gene Therapy
ISEV: International Society for Extracellular Vesicle
IVF: *In Vitro* Fertilization
LAL: Limulus amebocyte lysate
LPS: Lipopolysaccharide
MES: Manufacturing Execution Systems
MHC: Major Histocompatibility Complex
MSC: Mesenchymal Stromal Cell
NK: Nature Killer
NTA: Nanoparticle Tracking Analysis
PIC/S: The Pharmaceutical Inspection Convention and Co-operation Scheme
POC: Point-of-Care
QA: Quality Assurance
QC: Quality Control
RABS: Restricted Access Barrier System
SLE: Systemic Lupus Erythematosus
SOPs: Standard Operating Procedures
TEM: Transmission Electron Microscopy
VHP: Vapor Hydrogen Peroxide
WJ-MSC: Wharton’s Jelly-derived Mesenchymal Stromal Cell
